# Early and Late Results Following Choledochoduodenostomy and Choledochojejunostomy

**DOI:** 10.1155/1990/62814

**Published:** 1990

**Authors:** J. D. Blankensteijn, O. T. Terpstra

**Affiliations:** 1 Department of Surgery University Hospital “Dijkzigt” Erasmus University Rotterdam The Netherlands; 2 Department of Surgery University Hospital “Dijkzigt” Rotterdam 3015 GD The Netherlands

## Abstract

Objective —To evaluate the results and complications of choledochoduodenostomy and choledochojejunostomy for benign and malignant disease and to review them in the light of the survival of the underlying disorders.

Design —Retrospective analysis of medical records completed by a thorough inquiry for all patients who were lost to follow-up.

Setting —Referrals for primary and secondary surgery for obstructive biliary disease to a university hospital from 1974–1987.

Patients —After exclusion of patients who underwent a pancreaticoduodenectomy for cancer (Whipple procedure) 113 patients were included in the study (choledochoduodenostomy = CD, N = 64 and choledochojejunostomy = CJ, N = 49). A complete follow-up was achieved in 105 of 113 patients (93%).

Interventions —An inquiry was made at the civil registration office if the patients were alive or not. The general practitioners of the patients who had died were contacted about the cause of death and the possible biliary symptoms preceding death and the patients who were still alive received a questionaire which scrutinized all possible complications and side effects of the operation.

Endpoints —Cholangitis, recurrence of the underlying disease or death of the patient.

Measurements  and main results —Operative mortality was 4.7% following CD and 12.2% following CJ. Procedure-related complications were found in 10.9% and 28.6% respectively. Recurrent cholangitis was not seen after CD and in three patients with a CJ (6.1%). Survival following biliodigestive anastomosis for benign obstruction was comparable for age and sex matched survival.

Conclusions  —Although CD for choledocholithiasis has largely been replaced by endoscopic papillotomy and although the choice between the two procedures in malignant disease is most frequently dictated by the operative findings, we conclude that the choledochoduodenostomy is a relative simple operation with a low risk of cholangitis.


*HPB Surgery*, 1990, Vol. 2, pp. 151–158
Reprints available directly from the publisher
Photocopying permitted by license only

© 1990 Harwood Academic Publishers GmbH
Printed in the United Kingdom

### EARLY AND LATE RESULTS FOLLOWING CHOLEDOCHODUODENOSTOMY AND CHOLEDOCHOJEJUNOSTOMY

J.D. BLANKENSTEIJN, and O.T. TERPSTRA

*Department of Surgery, University Hospital "Dijkzigt", Erasmus University, Rotterdam, the Netherlands*

*(Received 29 May 1989)*

**Objective** — To evaluate the results and complications of choledochoduodenostomy and choledochojejunostomy for benign and malignant disease and to review them in the light of the survival of the underlying disorders.
**Design** — Retrospective analysis of medical records completed by a thorough inquiry for all patients who were lost to follow-up.
**Setting** — Referrals for primary and secondary surgery for obstructive biliary disease to a university hospital from 1974–1987.
**Patients** — After exclusion of patients who underwent a pancreaticoduodenectomy for cancer (Whipple procedure) 113 patients were included in the study (choledochoduodenostomy = CD, N = 64 and choledochojejunostomy = CJ, N = 49). A complete follow-up was achieved in 105 of 113 patients (93%).
**Interventions** — An inquiry was made at the civil registration office if the patients were alive or not. The general practitioners of the patients who had died were contacted about the cause of death and the possible biliary symptoms preceding death and the patients who were still alive received a questionaire which scrutinized all possible complications and side effects of the operation.
**Endpoints** — Cholangitis, recurrence of the underlying disease or death of the patient.
**Measurements and main results** — Operative mortality was 4.7% following CD and 12.2% following CJ. Procedure-related complications were found in 10.9% and 28.6% respectively. Recurrent cholangitis was not seen after CD and in three patients with a CJ (6.1%). Survival following biliodigestive anastomosis for benign obstruction was comparable for age and sex matched survival.
**Conclusions** — Although CD for choledocholithiasis has largely been replaced by endoscopic papillotomy and although the choice between the two procedures in malignant disease is most frequently dictated by the operative findings, we conclude that the choledochoduodenostomy is a relative simple operation with a low risk of cholangitis.

KEY WORDS: Biliary obstruction. Biliodigestive anastomosis. Choledochoduodenostomy. Choledochojejunostomy. Recurrent cholangitis.

Address for correspondence: J.D. Blankensteijn, MD Department of Surgery, University Hospital "Dijkzigt", 3015 GD Rotterdam, The Netherlands.

### INTRODUCTION

Over the last ten years endoscopic papillotomy reduced the number of patients who underwent surgery for bile duct stones and the procedure of choledochoduodenostomy (CD) disappeared from the surgical practice in most centers. Consequently choledochojejunostomy (CJ), which is used in cancer surgery, is preferred more and more, even in benign disease. If a biliodigestive anastomosis is indicated the choice between CD and CJ is usually determined by intraoperative considerations about the extension of tumour growth and the condition or the prognosis of the patient. There are, however, situations when both techniques are optional. For instance, in case of an impacted stone in the distal part of the common bile duct when preoperative endoscopic papillotomy was not performed or unsuccessful.

The question arises: which type of biliodigestive anastomosis is preferable when a choice is possible. Recurrent cholangitis, the most troublesome procedure related complication, has been reported to occur in 0.4–10% of the patients after CD and in 4.3–4.4% following CJ[1–5]. As only one report[2] comparing CD and CJ could be found, we retrospectively analyzed the results of the biliodigestive anastomoses performed in our hospital.

### PATIENTS AND METHODS

The medical records of all patients who underwent CD or CJ form January 1974 through January 1987 were reveiwed. Because of the specific nature of the procedure, the CJ after pancreaticoduodenectomy for cancer (Whipple procedure) was excluded from analysis. In this way 113 patients were included in the study: 56 males and 57 females with a mean age of 64.7 and 68.3 years respectively. From the operation report information about the type of anastomosis, the usage of a splint, the duration of the operation and the estimated quantity of blood loss was acquired.

In addition, with respect to the assessment of survival, the patients were retrospectively classified as to the indication for operation into three groups: stone formation, carcinoma and exploration (miscellaneous disease). These groups were established since survival is dependent on the type of the underlying disease. Therefore, in this report, mortality is considered from two viewpoints: the type of biliodigestive anastomosis and the indication for operation.

Techniques:

CD: A longitudinal incision of at least 1.5 cm length was made in the dilated portion of the common bile duct. After the Kocher manoeuver was carried out the duodenum was brought, without tension, adjacent to the choledochotomy and a transverse incision was made in the duodenum. The anastomosis was made by a running one-layer suture using absorbable material (catgut, Vicryl[R] or PDS[R]). Splinting of the anastomosis was used infrequently.

CJ: After opening the common bile duct as described above, an isolated jejunum loop in the Roux-en-Y fashion was created by dividing the jejunum approximately 15 cm distal to the Treitz ligament. The distal end was closed by continuous suture or staples and diverted through the mesentery of the transverse colon to the common bile duct. A side-to-side anastomosis between the bile duct and the jejunal loop at 2–3 cm from the closed end of the bowel was made with a simple running absorbable suture in one layer through the entire bowel wall. Again splints were seldom applied. An end-to-side jejunojejunostomy between the afferent small bowel and the Roux-en-Y loop completed the procedure.

Details about early and late complications and mortality were derived from the medical records. All patients who had not visited the outpatient clinic recently were verified to be alive at the civil registration office. With this information, at last follow-up, 72 patients had died and three could not be traced. The local physicians of the patients who had died were questioned about the cause of mortality and about symptoms of cholangitis preceding the death of the patient. Consequently, with regard to the surviving patients, an inquiry was conducted regarding the presence of abdominal pain, fever chills or episodes of jaundice. Questionnaires were sent to the remaining 38 patients and 33 were filled out correctly and returned (86.8% response rate). If answers suggested signs or symptoms of cholangitis in the past or present, patients were called to our clinic for verification of these symptoms and further evaluation. With this method we achieved a complete follow-up in 105 of 113 patients (93%) with a mean (SEM) of 34.4 (3.3) months (range: 9 days to 12 years) (62.5 (7.5) months for the patients who were alive at last follow-up).

Data were subjected to computerized statistical analysis. Discrete variables were analyzed by the chi-square test (Yate's continuity correction used when necessary). When two by two comparisons were made, the Fisher exact test was applied. Continuous variables were subjected to analysis of variance (Non- parametric tests used when necessary). Survival was assessed by life-table analysis as described by Kaplan and Meier[6] and compared with that expected in the Dutch population, corresponding in age, sex and year of diagnosis. Life-tables were compared using the log-rank test. All comparisons were two-tailed and $p < 0.05$ was considered significant.

### RESULTS

Choledochoduodenostomy was performed in 64 cases (30 males and 34 females, mean age 71.1 years) and choledochojejunostomy in 49 (26 males and 23 females, mean age 60.5 years). The duration of a CD-procedure was 120 7.2 minutes and of a CJ-procedure 165 7.5 minutes (median SEM). The estimated blood loss was 400 62.9 ml in the CD-group and 700 141.1 ml in the CJ-group. A splint was used in 11 patients (6/64 CD and 5/49 CJ). The operation was performed as an emergency operation in 10 out of 113 patients: seven with cholecystitis, cholangitis or gallstone-pancreatitis, one with acalculous cholecystitis, one with a complication of percutaneous transhepatic cholangiography and one with cholangitis after endoscopic papillotomy.

Stone formation was the underlying disease in 47 patients (41 CD and 6 CJ), carcinoma in 50 (12 CD and 38 CJ) and miscellaneous disease in 16 (11 CD and 5 CJ).

Most cases (eight) of the latter group were benign obstructions of the papilla, and in two cases the indication for operation was a iatrogenic choledochal duct lesion.

In 27 patients a cholecystectomy had been performed previously; in 81 patients the gallbladder was removed as an adjuvant to the biliodigestive anastomosis and in one patient an aplasia of the gallbladder was found, leaving only four patients who had a biliodigestive anastomosis with the gallbladder in situ; a meaningful evaluation regarding this condition could thus not be made.

*Early mortality and complications*

There were 9 deaths within thirty days of operation (9/113 = 8.0%): Three patients died after a CD (3/64 = 4.7%) and six after a CJ (6/49 = 12.2%). In the group of stone formation there was one death. This patient was operated upon because of a cholangitis that had followed a failed endoscopic papillotomy and he died after a period of septic shock, three weeks postoperatively. In the carcinoma-group six patients died: five directly related to the cancer and one from cardiac failure. In the miscellaneous-group two died: one from haemorrhagic shock and one from subphrenic abscess.

Following CD seven of 64 patients (10.9%) had eight complications which were related to the CD procedure: three bile leaks of which two sealed spontaneously, two haematoperitonea, two wound infections and one biliary obstruction. These complications led to two reexplorations and one related death (a patient with biliary obstruction and bile leakage). In 16 of the 64 patients (25%) with CD, 20 complications developed which were not related to the CD procedure: one bleeding gastric ulcer (this patient survived after a successful reoperation), two cardiac, three pulmonary, one thromboembolic and 13 other (minor) complications.

Following CJ 14 of 49 patients (28.6%) had 16 complications which were related to the CJ procedure. Five patients with postoperative bleeding were successfully reoperated. Two of these patients, however, died after 14 and 28 days respectively: one from preexisting liver cirrhosis and one from a further bleed caused by progression of the cancer. There were five bile leaks, of which four closed spontaneously, and of which one was reoperated because of an additional haematoperitoneum, four wound infections and two biliary obstructions. In 10 of the 49 patients (20.4%) with CJ, 11 complications developed which were not related to the CJ procedure: one bleed caused by progression of cancer, one cardiac, five pulmonary and four other (minor) complications. These data are summarized in Table 1.

*Long term results*

One hundred and four patients (46 stone formation, 44 carcinoma and 14 exploration) survived the 30-days postoperative period. At last follow-up 62 of these patients (61.4%) had died: 17 of 44 patients (38.6%) with stone formation, 39 of 43 (90.7%) with carcinoma and six of 14 (42.9%) explorations. The survival curves of the groups of stone formation and carcinoma are given in Figures 1 and 2. Each curve is accompanied by its corresponding expected survival curve of the Dutch population matched for age, sex and year of diagnosis. Survival following CD or CJ for biliary stones is not different from the expected matched survival, but in case of malignant disease this difference is remarkable.

*Recurrent cholangitis*

Recurrent cholangitis was found in none of 64 patients with CD and in three of 49 patients (6.1%) with CJ. The indication for the CJ in two of these three patients was stone formation and a choledochal cyst in one patient. Because the mortality in the CJ- group is considerably higher than in the CD-group, the average duration of the postoperative period over which a patient is exposed to the risk of developing cholangitis is also shorter in this study. Thus simple comparison of cholangitis-rates in both groups is inaccurate. Therefore the cumulative rates of recurrent cholangitis are compared according to the life-table method and the logrank test. This revealed the difference in cholangitis rate between the two groups of CD and CJ to be statistically significant (P = 0.0136).

There were no patients with pancreatitis after CD or CJ.

**Table 1** Summary of results and complications of choledochoduodenostomy (CD) and choledochojejunostomy (CJ).

| | *Type of anastomosis* *CD (N=64)* | *CJ (N=49)* | *Total (N=113)* |
|---|---|---|---|
| Early mortality*: | | | |
| N, % | 3, 4.7 | 6, 12.2 | 9, 8.0 |
| Procedure-related complications: | | | |
| N (N-complic), % | 7 (8), 10.9 | 14 (16), 28.6 | 21 (24), 18.6 |
| – Bile leak | 3 | 5 | 8 |
| – Haematoperitoneum | 2 | 5 | 7 |
| – Wound infection | 2 | 4 | 6 |
| – Biliary obstruction | 1 | 2 | 3 |
| Other complications: | | | |
| N (N-complic), % | 16 (20), 25.0 | 10 (11), 20.4 | 26 (31), 23.0 |
| – Pulmonary | 3 | 5 | 8 |
| – Cardiac | 2 | 1 | 3 |
| – Gastro-intestinal bleeding | 1 | 1 | 2 |
| – Thrombo-embolic | 1 | | 1 |
| – Other (minor) | 13 | 4 | 17 |

### DISCUSSION

Studies concerning the choice between the CD and CJ are scarce and, up until the time of writing, all retrospective[2,7–9]. Both procedures, however, appear to be effective for many comparable indications.

Obviously the two groups of CD and CJ in our study are not comparable. The difference in the number of malignancies as the indication for operation in the groups of CD and CJ is statistically significant: 12/64 CD (17.2%) and 38/49 CJ (37.5%) ($P<0.001$) as well as the difference in age between these two groups ($P<0.001$). Therefore data concerning the duration of the operations, the amount of blood loss, complications and early postoperative mortality are merely given for descriptive reasons. However, there can be no doubt about the fact that a CD-procedure is less extensive and less time-consuming.

The value of splinting the anastomosis is still controversial[10]. In case of thin-walled, not greatly dilated bile ducts it might prevent stenosis. Because in only 10% of our patients a splint was used, we could not analyze the possible benefits of this adjunct.

The fact that six CJ's were performed for stone formation and two CJ's for benign papillary obstructions confirms our concept that in some cases (less than 10%) a choice between the two types of anastomosis is possible.

The rates of complications which were related to the procedure are high: 10.9% for

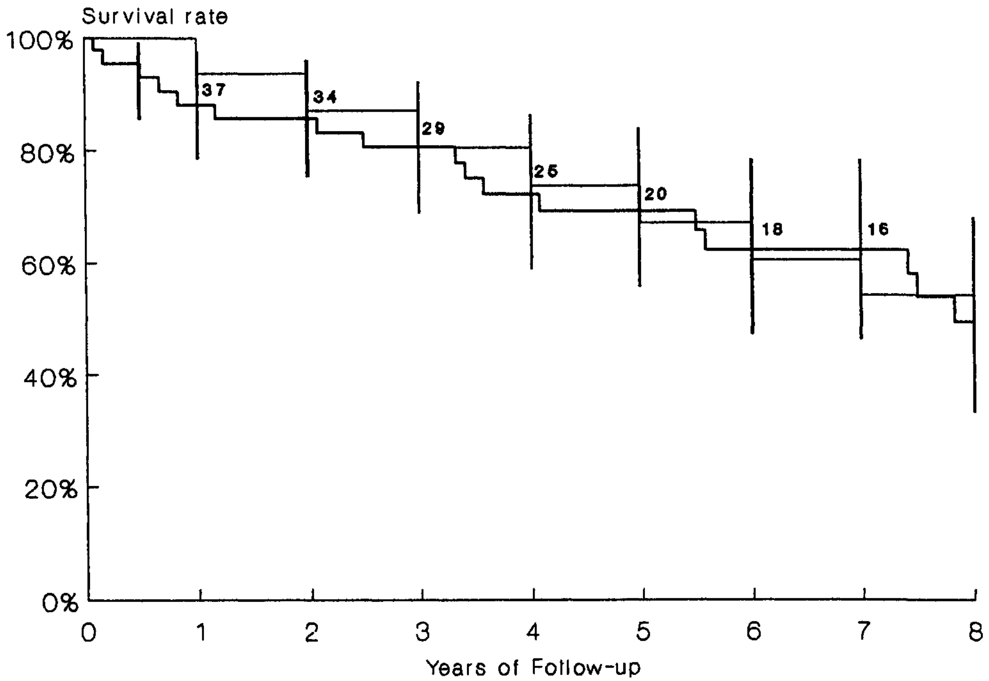

**Figure 1** Cumulative survival by life table analysis of 47 patients following a biliodigestive anastomosis for bile duct stones (bold curve) and the expected survival of the Dutch population matched for age, sex and year of diagnosis (thin curve). Vertical ranges are 95% confidence limits. Numbers are the patients at risk.

the CD-group and 28.6% for the CJ-group. This is consistent with earlier reports[1,4,9,10–13]. However, the varying indications for surgery and types of anastomoses in these reports as well as in our study, make the complication rates incomparable.

In an earlier report a correlation was suggested between the creation of a CJ and the development of a peptic ulcer, which may be related to the increased production of the Gastro-Intestinal Polypeptide Hormone[14]. This relation, however, is still controversial[3,15] and we could not confirm this finding as we only found one (bleeding) peptic ulcer following CD. We were surprised by finding no patients with recurrent cholangitis following CD despite our meticulous search.

Earlier studies reported a cholangitis rate of 2.6%-10% following a CD[2–4] but in a collected series of 1255 patients, the incidence of cholangitis was only 0.4%[5].

The two most important objections to CD are the "sump syndrome" and recurrent cholangitis. It is postulated that stones, sludge or food residue in the blind common bile duct pouch are held responsible for the development of cholangitis, jaundice or pancreatitis. However only few patients are actually described[16,17] and many authors could not identify similar problems[2,4,5].

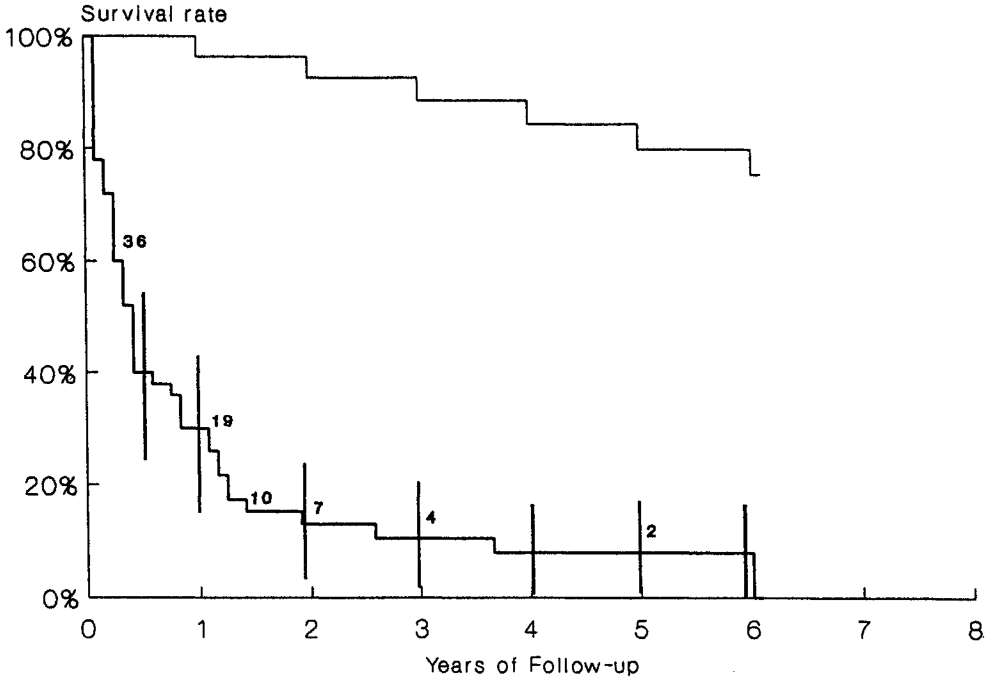

**Figure 2** Cumulative survival by life table analysis of 50 patients following a biliodigestive anastomosis for cancer (bold curve) and the expected survival of the Dutch population matched for age, sex and year of diagnosis (thin curve). Vertical ranges are 95% confidence limits. Numbers are the patients at risk.

Reflux of duodenal contents into the biliary system has been the presumed cause of recurrent cholangitis but experimental studies showed that cholangitis only developed in cases of a narrow or strictured anastomosis[5]. This finding is also of importance with regard to the etiology of the sump syndrome.

The poor survival of the CJ-group as compared to its expected matched survival and to the survival of the CD-group is explained by the fact that the former group consists largely of patients who were not suitable for curative resection of a malignant tumour of the pancreas, and in whom the CJ-procedure was performed for palliation.

In view of this poor survival (which is consistent with previous reports[9,11,18]) we believe that more attention should be given to palliative treatment of (impending) biliary obstruction by carcinoma of the head of the pancreas. Results of radiotherapy and chemotherapy are modest at best[9]. In case a decision about the type of anastomosis is to be made, it is essential to consider the disappointing cumulative survival of 30% at one year. Effective bile drainage using endoscopic techniques of choledochoduodenostomy[19] and endoprothesis placement[20], with lesser procedure related complications as compared to biliodigestive anastomoses, have been described. Not surprisingly many patients with obsruction of the distal portion of the common bile duct are treated by endoscopic techniques nowadays or other non-invasive procedures like lithotripsy or dissolution of stones.

From our study we conclude that CD is far from obsolete in the treatment of common bile duct obstruction and still deserves it place in hepatobiliary surgery. It is a relatively simple operation and even though reflux of duodenal contents into the bile duct system may be considerable, it has low risk of cholangitis.

*References*

1. Bismuth, H, Franco, D, Corlette, M.B., Hepp, J. Long term results of Roux-en-Y hepaticojejunostomy. *Surg. Gynecol. Obstet* .1978; **146,** 161–7.
2. Vogt , D.P., Hermann, R.E. Choledochuduodenostomy, choledochojejunostomy or sphincteroplasty for biliary and pancreatic disease. *Ann. Surg* .1981; **193:,** 161–8.
3. Anderberg, B., Bolin, S., Heuman, R., Sjödahl R. Choledochoduodenostomy for choledocholithiasis. Indications and functional results. *Acta Chir Scand* 1984; **150:,** 75–8.
4. Huguier , M., Lacaine, F., Houry , S., Pascal , G. Choledochoduodenostomy for calculous biliary tract diseas. *Arch. Surg* .1985; **120:,** 241–2.
5. Madden, J.L., Chun, J.Y,. Kandalaft S, Parekh M. Choledochoduodenostomy — an unjustly maligned surgical procedure. *Am. J. Surg.* 1970; **119:,** 45–54.
6. Kaplan, E.L., Meier, E.A. Nonparametric estimation from incomplete observations. *J. .Am. Statistical Assoc* . 1958; **53:** ,457–81.
7. Fry , D.E. Surgical techniques in the management of distal biliary tract obstruction. *Am. Surg.* 1983; **49:,** 138–42.
8. Tondelli, P., Ackermann, Ch, Blumgart, L.H. Bilio-digestive Anastomosen bei benignen Gallenwegserkrankungen.. *Chirurg.* 1984; **55,:** 777–86.
9. Schouten, J.T. Operative therapy for pancreatic carcinoma. *Am. J .Surg* .1986; **151,:** 626–30.
10. Genest, J.F., Nanos, E., Grundfest-Broniatowsky S., Vogt , D., Hermann, R.E. Benign biliary strictures: An analytic review (1970 to 1984). *Surgery* 1986; **99:** ,4 09–13.
11. Sonnenfeld, T., Nyberg ,B., Perbeck, L. The effect of palliative biliodigestive operations for unresectable pancreatic cancer. *Acta.. Chir. Scand.* 1986, suppl; **530:,** 47–50.
12. Lygidakis ,N.J. Choledochoduodenostomy in calculous biliary tract disease. *Br .J .Surg.* 1981; **68:** 762– 5.
13. Birkenfeld, S., Serour, F., Levi . S., Abulafia, A., Balassiano, M., Krispin. M. Choledochoduodenostomy for benign and malignant biliary tract diseases. *Surg* .1988; **103:,** 408–410.
14. Sato, T., Imamura .M., Sasaki , I., Kameyama, J. Gastric acid secretion and gastrointestinal hormone release after biliary reconstruction procedures. *Am. J. Surg* .1983; **146:** 245–9.
15. Stefanini , P., Carboni, N., Petrassi, N. Roux-en-Y- hepaticojejunostomy: A reappraisal of its indications and results. *Ann. Surg* .1975; **181:** 213–6.
16. Tondelli, P., Gyr, K. Biliary tract disorders: Postsurgical syndromes. *Clin. Gastroenterol.* 1983; **12:** 231–8.
17. Rutledge, R.H. Sphincteroplasty and choledochoduodenostomy for benign biliary obstructions. *Ann. Surg* .1976; **183:** 476–87.
18. Nakase, A., Matsumoto, Y., Uchida, K., Honjo, I. Surgical treatment of the pancreas and periampullary region. Cumulative results in 57 institutions in Japan. *Ann. Surg.* 1976; **183:** 52–7.
19. Aabakken, L., Osnes, M. Endoscopic choledochoduodenostomy (ECDT) as palliative treatment of malignant periampullary obstructions of the common bile duct: a follow-up study. *Gastrointest. Endosc* . 1986; **32:** 41–2.
20. Huibregtse, K., Katon, R.M., Coene, P.P., Tytgat, G.N.J. Endoscopic palliative treatment in pancreatic cancer. *Gastrointest. Endosc.* 1986; **32:** 334–8.

*(Accepted by S. Bengmark 29 May 1989)*

